# Interactive effects of solar radiation and dissolved organic matter on bacterial activity and community structure

**DOI:** 10.1111/j.1462-2920.2007.01334.x

**Published:** 2007-09

**Authors:** María Teresa Pérez, Ruben Sommaruga

**Affiliations:** Laboratory of Aquatic Photobiology and Plankton Ecology, Institute of Ecology, University of Innsbruck Technikerstrasse 25, 6020 Innsbruck, Austria.

## Abstract

We studied the interactive effects of dissolved organic matter (DOM) and solar radiation on the activity and community structure of bacteria from an alpine lake. Activity was assessed both at the community level as leucine incorporation rates and at the single-cell level by microautoradiography. Fluorescent *in situ* hybridization and signal amplification by catalysed reporter deposition (CARD-FISH) was used to track changes in the bacterial community composition. Bacteria-free filtrates of different DOM sources (lake, algae or soil) were incubated either in the dark or exposed to solar radiation. Afterwards, the natural bacterial assemblage was inoculated and the cultures incubated in the dark for 24–48 h. Bacterial activity was enhanced in the first 24 h in the soil and algal DOM amendments kept in the dark. After 48 h, the enhancement effect was greatly reduced. The initial bacterial community was dominated by *Betaproteobacteria* followed by *Actinobacteria*. The relative abundance (expressed as a percentage of DAPI-stained cells) of *Betaproteobacteria* increased first in dark incubated DOM amendments, but after 48 h no significant differences were detected among treatments. In contrast, the relative abundance of *Actinobacteria* increased in pre-irradiated DOM treatments. Although *Betaproteobacteria* dominated at the end of the experiment, the relative abundance of their R-BT subgroup differed among treatments. Changes in bacterial community activity were significantly correlated with those of the relative abundance and activity of *Betaproteobacteria*, whereas the contribution of *Actinobacteria* to the bulk activity was very modest. Our results indicate a negative effect of DOM photoalteration on the bulk bacterial activity. The magnitude of this effect was time-dependent and related to rapid changes in the bacterial assemblage composition.

## Introduction

There is substantial evidence that dissolved organic matter (DOM) exhibits photoreactive properties particularly, when exposed to radiation in the UV-B (280–320 nm) and UV-A (320–400 nm) range. Photoproducts derived from DOM upon sunlight exposure can both enhance (Reitner *et al*., 1997; De Lange *et al*., 2003) and inhibit bacterioplankton activity (Tranvik and Kokalj, 1998; Pausz and Herndl, 1999), depending on the age and source of the DOM (Benner and Biddanda, 1998; Obernosterer *et al*., 1999; Tranvik and Bertilsson, 2001).

In aquatic ecosystems, the DOM fuelling bacterial metabolism can be classified according to its origin as autochthonous or allochthonous. Autochthonous DOM is by definition produced within the system and derives largely from primary producers, whereas allochthonous DOM originates externally mainly in the surrounding terrestrial ecosystem. These two DOM types differ in their chemical and optical characteristics (McKnight *et al*., 1994; 2001; Benner, 2002), upon which depend their fate when exposed to solar radiation. Although recent studies have shown that the DOM quality influences the composition and possibly the functioning of the bacterial assemblage (Judd *et al*., 2006; Kritzberg *et al*., 2006; Pérez and Sommaruga, 2006), there is no information available on how photochemically altered DOM affects the structure of the bacterial community and the activity of particular bacterial groups.

Typically, *Betaproteobacteria* numerically dominate the heterotrophic bacterial assemblage in freshwater ecosystems, followed by *Actinobacteria* (Glöckner *et al*., 2000; Burkert *et al*., 2003; Hahn *et al*., 2003), which are particularly abundant in alpine lakes at high altitude (Warnecke *et al*., 2005). The *Cytophaga*-like bacteria are numerically important in both freshwater and marine bacterial communities (Kirchman, 2002). Among the marine bacterial assemblage it has been shown that different phylogenetic groups differ in their ability to utilize specific dissolved organic compounds (Cottrell and Kirchman, 2000; Elifantz *et al*., 2005; Malmstrom *et al*., 2005). Although no similar study has been performed in freshwater ecosystems, these results suggest that changes in the DOM composition (i.e. related to its origin or due to photochemical transformation) might affect the bacterial community structure and/or activity.

Alpine lakes (i.e. located above the treeline) are relevant ecosystems to study DOM and solar radiation interactions because they receive higher instantaneous solar UV fluxes than lowland ones due to the increase of UV radiation with altitude and are highly transparent to this radiation (Laurion *et al*., 2000; Sommaruga and Augustin, 2006).

The pronounced air warming in the Alps (Beniston, 2000) might lead to shorter ice-cover periods at times of intense solar radiation, but might as well trigger a change in the quality of substrates fuelling the microbial compartment of the lake by enhancing the development of vegetation and soils in the catchment (Hauer *et al*., 1997).

In this study, we present results from two experiments conducted to test the combined effects of DOM and solar radiation on the composition and activity of the bacterial assemblage from the alpine lake Gossenköllesee (GKS). For this test, we used DOM derived from three different sources: the lake (control), an algal lysate and a soil extract (from GKS catchment), as respective autochthonous and allochthonous DOM surrogates. Because the simulation of the natural solar spectrum is challenging, the different DOM sources were either exposed to natural (first experiment) or simulated solar radiation (second experiment) to compare the response of the DOM to both radiation sources. Furthermore, in the first experiment we assessed changes in bulk bacterial activity, whereas in the second one, we additionally followed changes in the structure of the bacterial community and in the activity of specific bacterial groups.

## Results

### DOM characteristics

The elemental composition of the DOM used as a control (i.e. lake water) as well as that of the algal and soil amendments is summarized in Table 1. This table includes data from the second experiment, but it is representative of the first experiment as well, as the same algal and soil extracts were used in both experiments and the lake DOM, used as control, did not exhibit any substantial change between both experiments. The DOC concentration in both DOM amendments was ∼2.5-fold that of the control treatment. The different DOM sources were relatively similar in their C : N molar ratio, although the lowest ratio corresponded to the algal-derived DOM. The C : P ratio in the algal treatment was between 4.8 and 3.4 times lower than the one of the control and the soil amended treatment. In both experiments, the *a*_250_ : *a*_365_ ratio increased in all treatments upon irradiation (Table 2), indicating an increase of the relative proportion of low molecular size material. The absorption coefficient at 254 nm (*a*_254_) increased upon irradiation in all treatments, whereas the absorption coefficient at 320 nm (*a*_320_) did only increase in the pre-irradiated control DOM from experiment 2. The DOC-specific absorption coefficients at 254 nm (a*_254_) ranged from 0.010 to 0.014 l μmol DOC^−1^ m^−1^ in dark samples, except for the dark control (0.022 l μmol DOC^−1^ m^−1^) from experiment 1. Upon irradiation, the a*_254_ increased by 1.5- to 2.5-fold depending on the DOM type and experiment considered (data not shown).

**Table 1:** Elemental composition and molar ratios of the DOM in the original lake water (control), and in the algal- and soil-derived DOM immediately after their addition.

Treatment	TDP (μM)	DON (μM)	DOC (μM)	C : N (molar)	C : P (molar)
Control	0.05	6.15	57.4	9.35	1188
Algal	0.61	21.1	152.5	7.23	249
Soil	0.17	11.0	143.3	13.0	843

TDP, total dissolved phosphorus; DON, dissolved organic nitrogen. Please note that the C : P ratio is based on TDP concentrations that include a not determined fraction of inorganic phosphorus. Nutrient analyses were performed as described by Psenner (1989).

**Table 2:** Optical characteristics of the DOM used in both experiments.

	Experiment 1								Experiment 2							
	DOC		*a*_250_/*a*_365_		*a*_254_		*a*_320_		DOC		*a*_250_/*a*_365_		*a*_254_		*a*_320_	
	DK	IR	DK	IR	DK	IR	DK	IR	DK	IR	DK	IR	DK	IR	DK	IR
Control	53.2 ± 1.96	52.6 ± 1.72	2.80 ± 0.06	4.72 ± 0.08	1.15 ± 0.07	2.54 ± 0.05	0.41 ± 0.07	0.39 ± 0.03	57.4 ± 3.16	59.9 ± 3.86	1.82 ± 0.25	3.21 ± 0.39	0.80 ± 0.08	1.86 ± 0.29	0.42 ± 0.07	0.58 ± 0.02
Algal	118 ± 9.42	112 ± 4.20	3.03 ± 0.52	4.15 ± 0.15	1.44 ± 0.15	3.51 ± 0.75	0.69 ± 0.13	0.68 ± 0.11	153 ± 3.07	150 ± 5.37	2.57 ± 0.18	3.72 ± 0.09	1.52 ±0.01	2.28 ± 0.10	0.78 ± 0.02	0.76 ± 0.06
Soil	121 ± 6.65	116 ± 9.54	2.96 ± 0.14	4.61 ± 0.21	1.71 ± 0.06	4.10 ± 0.79	0.82 ± 0.05	0.74 ± 0.02	143 ± 0.79	142 ± 2.67	2.54 ± 0.17	4.11 ± 0.37	2.04 ± 0.07	3.02 ± 0.26	0.99 ± 0.00	0.97 ± 0.05

DOC concentrations are in μM. The absorption coefficients at 254 nm and 320 nm (*a*_254_ and *a*_320_) are expressed in m^−1^. DK, dark exposure; IR, irradiated. Values are mean of three replicates ± 1 SD.

### Bacterial abundance

**Fig. 1:**
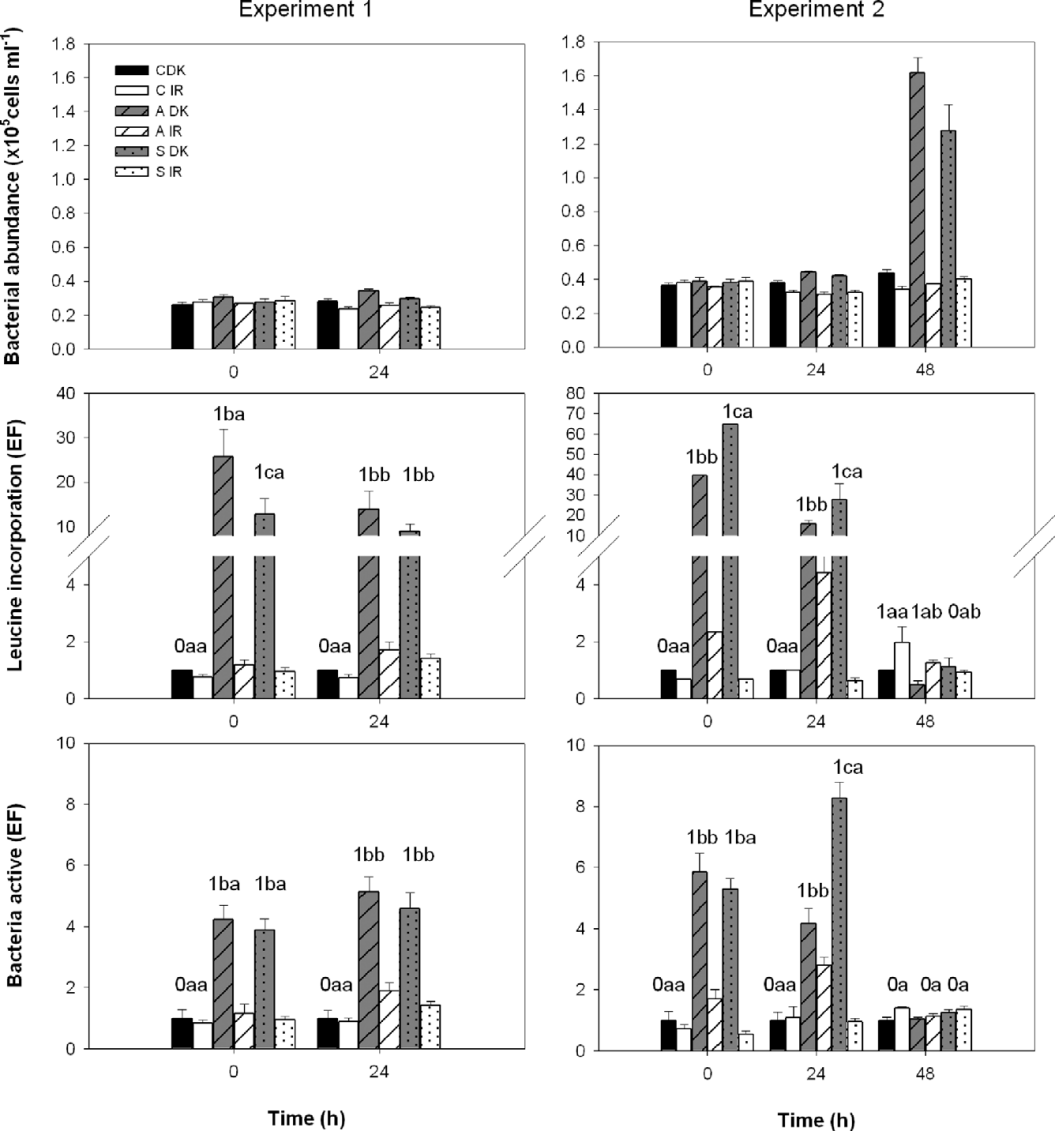
Temporal changes in bacterial abundance (top), bulk leucine incorporation (middle) and active bacteria (bottom) during the experiment with natural (left) and simulated solar radiation (right). Activity parameters are expressed as enhancement factor (EF = leucine incorporation rates or percentage of active cells in a given treatment versus leucine incorporation rates or percentage of active cells in the dark control). The first letter in the legend code corresponds to the DOM type: C is control, A is algal-derived DOM and S is soil-derived DOM, whereas the last two letters refer to the exposure conditions, either dark (DK) or pre-irradiated (IR). The number and letter code above the bars summarizes the results of the *post hoc* all pairwise multiple comparison test used to detect significant differences among DOM types and exposure conditions. The number indicates a significant (1) or non-significant (0) difference between exposure conditions for a given DOM type, whereas a different letter indicates a significant difference among DOM types at a given exposure condition. Comparisons were performed by the Holm–Sidak method with an overall significance level of 0.05. Values are mean of three replicates ± 1 SD.

Bacterial abundance in the initial samples ranged from 2.58 to 3.61 × 10^4^ cells ml^−1^ depending on the experiment considered (Fig. 1, top). After 24 h, bacterial abundance slightly increased in all dark samples, whereas it did not change or slightly decreased in the pre-irradiated ones. In the second experiment after 48 h, bacterial numbers in the algal- and soil-DOM amendments kept in the dark increased approximately fourfold, whereas they remained close to their initial value in the pre-irradiated treatments.

### Leucine incorporation and bacteria active

Within 24 h, no significant differences in bacterial activity (expressed as either bulk leucine incorporation or active bacteria estimated by microautoradiography) were detected between the dark and pre-irradiated control in both experiments (Fig. 1). Leucine incorporation was only significantly enhanced in the pre-irradiated control at 48 h in the second experiment. During the initial 24 h, leucine incorporation and the percentage of active bacteria were significantly enhanced in the dark samples of both DOM amendments (soil and algal) as compared with the control and their respective pre-irradiated counterparts. In the second experiment, the enhancement factor for both leucine incorporation and the amount of active bacteria was greatly reduced at 48 h.

### Community structure and activity of specific bacterial groups

Between 82% and 96% of the DAPI-stained cells were detected with probe EUB338 throughout the experiment. The filtration used to set up the experiment did not affect the relative abundance of *Betaproteobacteria* and *Actinobacteria*, but reduced the amount of filaments belonging to the *Cytophaga*-like bacteria, which were anyway present in very low proportion (1–2% of DAPI-stained cells). The bacterial community structure at the beginning of the experiment was dominated by *Betaproteobacteria* (∼50% of DAPI-stained cells), followed by *Actinobacteria* (∼27% of DAPI counts). The R-BT subgroup of *Betaproteobacteria* represented ∼20% of DAPI counts. Results with the probe CF319a targeting the *Cytophaga*-like bacteria were not included because it detected < 1% of DAPI-stained cells and thus, the relative abundance of this group could not be estimated accurately.

**Fig. 2:**
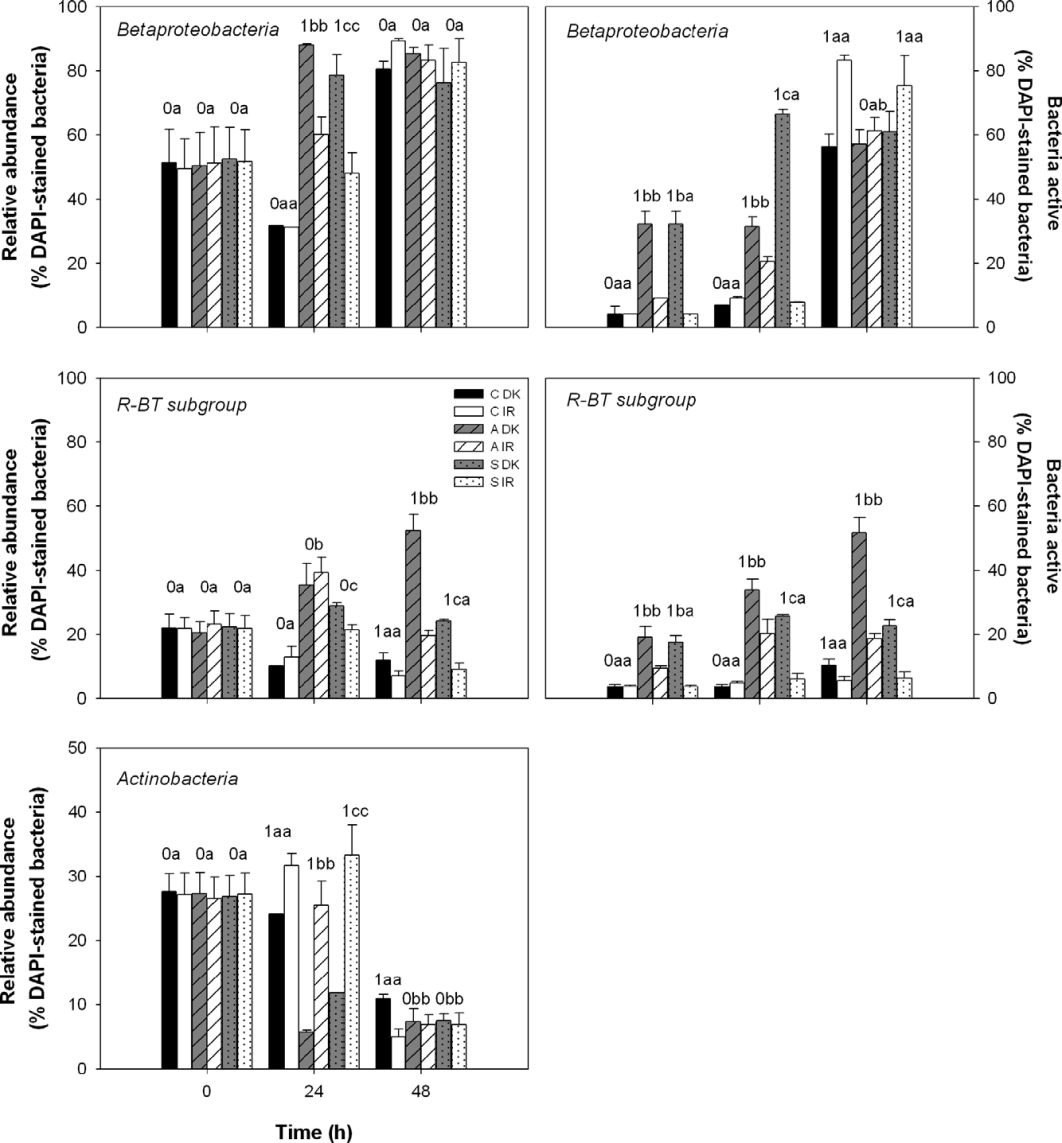
Temporal changes in the relative abundance (left) and single-cell activity (right) of *Betaproteobacteria* (top), the R-BT subgroup of *Betaproteobacteria* (middle) and *Actinobacteria* (bottom) during experiment 2. The first letter in the legend code corresponds to the DOM type: C is control, A is algal-derived DOM and S is soil-derived DOM, whereas the last two letters refer to the exposure conditions, either dark (DK) or pre-irradiated (IR). The number and letter code above the bars summarizes the results of the *post hoc* all pairwise multiple comparison test used to detect significant differences among DOM types and exposure conditions. The number indicates a significant (1) or non-significant (0) difference between exposure conditions for a given DOM type, whereas a different letter indicates a significant difference among DOM types at a given exposure condition. Comparisons were performed by the Holm–Sidak method with an overall significance level of 0.05. Values are mean of three replicates ± 1 SD.

The relative abundance of *Betaproteobacteria* decreased in both control samples after 24 h (Fig. 2, top), whereas it remained high or even increased in both DOM amendments regardless of the exposure conditions. However, within a DOM amendment, *Betaproteobacteria* relative abundance was significantly higher in the samples kept in the dark as compared with the pre-irradiated ones. After 48 h, no significant differences in the relative abundance of this group neither due to the DOM type nor to the exposure conditions were detected.

Immediately after inoculation (0.5 h), the proportion of active *Betaproteobacteria* in the control was low (Fig. 2, top). A significant effect of the DOM exposure conditions on *Betaproteobacteria* activity was observed, and higher relative abundances were found in both DOM amendments kept in the dark as compared with the pre-irradiated ones. This pattern was still observed at 24 h and was particularly pronounced in the soil DOM amendment. At 48 h, the relative abundance of active *Betaproteobacteria* was high in all treatments and in the pre-irradiated control significantly higher as in its dark counterpart.

The relative abundance of the R-BT subgroup of *Betaproteobacteria* (Fig. 2, middle) did slightly decrease after 24 h in the dark and pre-irradiated control, whereas it increased in the treatments amended with either the algal- or soil-derived DOM. No significant differences in the relative abundance of this subgroup were found between the dark and pre-irradiated samples, regardless of the DOM type. However, there was a significant effect of the DOM source on the R-BT subgroup that resulted in higher relative abundances in the algal-derived DOM, as compared with the soil-derived DOM and the control. At 48 h, the relative abundance of R-BT bacteria was always significantly higher in the dark samples as compared with the pre-irradiated ones, not only in the DOM amendments but also in the control. Furthermore, at 48 h pre-irradiated algal-derived DOM had a significant positive effect on the relative abundance of R-BT bacteria as compared with the pre-irradiated control.

At the beginning of the experiment, the percentage of active R-BT cells (Fig. 2, middle) was low in the control samples. Both dark incubated DOM amendments had a significant positive effect on the activity of the R-BT subgroup. The relative abundance of active R-BT cells in pre-irradiated algal-derived DOM was significantly higher than in the pre-irradiated control and soil-derived DOM. At 24 h, the proportion of active R-BT bacteria significantly increased in algal-derived DOM-amended samples. Dissolved organic matter amendments incubated in the dark sustained a higher fraction of active R-BT bacteria than their irradiated counterparts. The same trend was observed at 48 h, but the differences between dark and pre-irradiated samples were more pronounced.

*Actinobacteria* represented ∼27% of the DAPI counts (Fig. 2, bottom) at the beginning of the experiment. After 24 h, the relative abundance of this group significantly differed between the dark and pre-irradiated samples in the control as well as in both DOM amendments. The relative abundance of *Actinobacteria* remained at their initial level or even increased in pre-irradiated DOM, whereas it decreased in the soil- and algal-derived DOM amendments incubated in the dark. At 48 h, a decrease in the relative abundance of *Actinobacteria* was apparent in all treatments. *Actinobacteria* contributed very modestly to total activity (data not shown) with < 2% of total counts corresponding to active cells at the beginning of the incubation.

## Discussion

Our work confirms the results of some previous studies on photochemically induced changes in DOM and their subsequent effects for bacterial growth, but it also provides a novel insight on how the bacterial community composition and the activity of particular bacterial groups is affected.

### Photoalteration of the DOM and its effects on bacterial activity

Results from previous studies suggest that the effects of solar radiation on DOM availability for bacteria depend to a larger extent on DOM characteristics such as its age and origin (Benner and Biddanda, 1998; Obernosterer *et al*., 1999; Tranvik and Bertilsson, 2001). Thus, it has been argued that freshly produced or autochthonous DOM is likely to become more recalcitrant for bacteria upon sunlight exposure (Tranvik and Kokalj, 1998; Pausz and Herndl, 1999), whereas in contrast, old or allochthonous DOM enhances bacterial growth upon irradiation (Benner and Biddanda, 1998; Anesio and Granéli, 2003). In our experiments, however, both DOM types (algal and soil) led to a decrease of the bulk bacterial activity and of the percentage of active bacteria when pre-exposed to natural or simulated solar radiation. Whereas a negative effect on bacterial activity was expected in the treatment receiving pre-irradiated algal-derived DOM, the decrease of bacterial activity in pre-irradiated soil-derived DOM was not. Terrestrially derived DOM is generally considered refractory to bacterial degradation (Del Giorgio and Davis, 2003) because of its high content in humic substances and structural polysaccharides (Benner, 2002; 2003). Thus, it is supposed to become more bioavailable upon sunlight exposure (Smith and Benner, 2005). However, the initial optical characteristics of algal- and soil-derived DOM (Table 2) were very similar and both DOM types behaved in an analogous way after exposure to solar radiation. Particularly, the calculation of the DOC-specific absorption coefficient at 254 nm (a*_254_), which is used as a proxy for the degree of aromaticity of organic matter (Weishaar *et al*., 2003), indicated a low degree of aromaticity in both DOM types, which was comparable to that of the lake DOM. Size-exclusion gel chromatography of DOM from GKS has revealed that the largest DOC fraction in this alpine lake does not significantly absorb UV radiation (Sommaruga and Augustin 2006). The increase in a*_254_ upon irradiation of all DOM treatments suggested a humification process. Absorption coefficients at 254 nm in irradiated DOM samples were often double as high as those measured in DOM kept in the dark, whereas the DOC concentrations remained fairly stable. Brisco and Ziegler (2004) proposed a similar process to explain the observed decrease in the bioavailability of relatively refractory organic matter following exposure to solar radiation.

One plausible explanation for the unexpected ‘lability’ of the soil extract could lay in the procedure we used to obtain it (Kablitz *et al*., 2003; Pérez and Sommaruga, 2006). Basically, we simulated the effects of runoff and, therefore, extracted the soil organic matter (SOM) in Milli-Q water at *in situ* temperature. By doing so, it is very likely that only the more labile fraction of SOM was extracted, as the extraction of humic acids needs a certain degree of alkalinity. Moreover, it is arguable that SOM from alpine catchments located at high altitude that undergoes low microbial degradation because of low temperatures (Leifeld *et al*., 2005) has still a substantial labile organic fraction available.

### Effects on the bacterial community structure and the activity of bacterial groups

We proved that the photoalteration of DOM not only affected its subsequent utilization by bacteria, but it also translated into changes in the bacterial community structure. Different bacterial groups showed a distinct response to the different substrates offered. Moreover, we observed a contrasting effect on bacterial activity parameters depending on the incubation time considered. Effects on leucine incorporation and on the relative abundance of active bacteria were noticeable immediately after inoculation (0.5 h) of the bacterial assemblage. It is remarkable that the activation of possibly ‘starved’ bacteria (Morita, 1982) took place almost instantaneously and when an appropriate substrate was offered the relative abundance of active bacteria increased from 4% to 32% in only half an hour. The enhancement of bacterial activity in the dark treatments was maintained at 24 h; however, after 48 h this effect was reduced or in some cases even reversed. Bertilsson and colleagues (2004) found an initial decrease in bacterial production in irradiated South Ocean water that was not detectable in a longer incubation. These authors hypothesized that either the pool of the most rapidly available compounds was photochemically transformed into biorecalcitrant material or photochemical processes have a minor impact on bacterial activity at low temperatures. We cannot rely on the second hypothesis because our experiments were run at 14°C which is at the upper water temperature limit in GKS. However, changes in DOM optical characteristics indicating a humification process favoured the first hypothesis. Besides that, in our experiments, the contrasting effect on bacterial activity was accompanied by changes in the relative abundance of particular bacterial groups. After 24 h, there was a distinct effect on the bacterial community composition. On one hand the *Betaproteobacteria* were stimulated in both DOM amendments kept in the dark. On the other hand, the relative abundance of *Actinobacteria* increased in the pre-irradiated DOM and decreased sharply in the algal- and soil-derived DOM kept in the dark as compared with the dark control (lake DOM). The contrasting behaviour of these two bacterial groups was supported by a highly significant negative relationship (*r*^2^ = 0.52; *P* < 0.0001), when data from all treatments were pooled. Such a strong negative relationship between these two groups has already been found during an experimental manipulation of the bacterial assemblage of GKS (Pérez and Sommaruga, 2006) and that of Lake Fuchskuhle, Germany (Burkert *et al*., 2003). This negative relationship is also in agreement with the finding that in GKS there is a temporal segregation in the maximum abundance of these two groups (Glöckner *et al*., 2000). Thus, we suggest that these two bacterial groups occupy similar niches, but *Actinobacteria* are easily outcompeted by *Betaproteobacteria*. There is to our knowledge no similar study in which the response of bacterial groups to photoaltered DOM has been assessed, but our data suggest that *Actinobacteria* could be favoured under those circumstances. In fact, the percentage of *Actinobacteria* has been found to increase within the bacterial community of mountain lakes, particularly of those located at high altitude (Warnecke *et al*., 2005), where DOM photoalteration could be an important process.

The abundance of the R-BT subgroup of *Betaproteobacteria* was not affected by the exposure conditions at 24 h, but only by the origin of the DOM, and showed a marked preference for algal-derived DOM. A similar positive effect of algal-derived DOM on the R-BT subgroup was observed in our previous work (Pérez and Sommaruga, 2006) but also in the mesotrophic Øimov Reservoir, Czech Republic, when microcosms were manipulated with inorganic nutrients (Šimek *et al*., 2005).

In terms of activity, the pattern observed for the whole bacterial community was mainly driven by the response of *Betaproteobacteria*. Highly significant correlations were found between the relative abundance of this particular group and the activity parameters (*r*^2^ = 0.53 with leucine incorporation rates and *r*^2^ = 0.72 with percentage of active bacteria; for both *P* < 0.0001). Theses correlations were even stronger when we considered only the fraction of active *Betaproteobacteria* (*r*^2^ = 0.74 and *r*^2^ = 0.98 for leucine incorporation rates and percentage of active bacteria, respectively; for both *P* < 0.0001). Although *Actinobacteria* was the second numerically important bacterial group, their contribution to activity was generally < 2% total counts. Their modest contribution to activity might be partly due to the method used to assess the percentage of active bacteria in our experiments. As explained in the *Experimental procedures*, we performed microautoradiography using leucine as substrate and incubated our samples for 1 h. Whereas a short incubation is appropriate to determine the relative contribution of different groups to the leucine incorporation rates we measured, it might bias the number of active bacteria for bacterial groups with a lower maximum velocity (*v*_max_). In fact, in a previous study (Pérez and Sommaruga, 2006) a larger contribution of *Actinobacteria* to the total activity was detected using a longer incubation period.

At the end of the experiment, *Betaproteobacteria* dominated all treatments coinciding with an attenuation of differences in bacterial activity in the different DOM types. Nevertheless, different *Betaproteobacteria* subgroups grew in the different treatments as indicated by the variable contribution of the R-BT bacteria. This subgroup represented > 50% of DAPI counts in the algal-derived DOM kept in the dark, whereas in the pre-irradiated lake water and soil-derived DOM treatment, their contribution was < 10% DAPI-stained cells. As previously stated, the R-BT subgroup was favoured in algal DOM kept in the dark, whereas one or several unknown *Betaproteobacteria* phylotypes were stimulated in photoaltered DOM. These unknown *Betaproteobacteria* did not belong to the Beta II lineage of limnic *Betaproteobacteria* (Bet2-870; M.T. Pérez, pers. obs.) described by Burkert and colleagues (2003). In mountain lakes, the fraction of *Betaproteobacteria* that are not targeted by currently available probes is not negligible (Warnecke *et al*., 2005).

In summary, our experiments showed that the effects of DOM photoalteration on the activity of the bacterial assemblage depend on the type of DOM present and are not always easily predictable from simple criteria as its origin. Photochemically induced changes in DOM had an effect on the bacterial community as a whole, but also affected individual bacterial groups. The magnitude of this effect was time-dependent and related to rapid changes in bacterial community composition.

## Experimental procedures

### Study site

Gossenköllesee is a small (area: 0.017 km^2^) alpine lake located at 2417 m above sea level in the Austrian Alps (47°13′N, 11°01′E). Gossenköllesee is a dimictic and holomictic lake covered by ice for about 7–8 months per year. The catchment area is composed of crystalline bedrock and covered with a poor soil layer and sparse patches of alpine rankers. Background information on DOM dynamics, chemical composition and other variables is found elsewhere (Sommaruga and Augustin, 2006).

### Experimental design

Two experiments were conducted during August 2005 using lake water collected from GKS at 2 m depth. The lake water was gently filtered first through a pre-combusted glass-fibre filter (AP 40, Millipore) and subsequently through a 0.22 μm polycarbonate membrane (GTTP, Millipore) to eliminate bacteria. Filtered water was then distributed among three sets of six replicate quartz tubes (250 ml; diameter: 5 cm). The first set of tubes was not further manipulated and served as control. The lake water in the second set of tubes was amended with a soil extract (soil-derived DOM treatment), whereas the third set received an algal lysate to enrich the autochthonous fraction of DOM (algal treatment). The soil extract was obtained according to Kablitz and colleagues (2003) using surface soil (upper 3–4 cm) collected from the catchment area of GKS. The lysate was obtained from a batch culture of the planktonic green algae *Chlorella minutissima* grown in Woods Hole medium (at 17°C, 8:16 light : dark cycle) and harvested in the early stationary phase, in order to allow the inorganic nutrients to be mostly incorporated by the algae. Prior to use, both extracts were filtered through a 0.22 μm polycarbonate membrane to eliminate bacteria and debris. Soil and algal DOM amendments enriched by a factor of 2–3 the DOC concentration as compared with the control. Then, half of the quartz tubes were wrapped in a double layer of aluminum foil, whereas the other half was not. During the first experiment, the tubes were exposed horizontally at the surface of GKS during 3 sunny days. For the second experiment, the exposure took place during 6 h in a walk-in room set at 14°C. Simulated solar UVR (8.60 W m^−2^ UVA and 2.47 W m^−2^ UVB) was provided by four aged (100 h) fluorescent lamps (UVA 340, Q-panel, Cleveland, OH) and two visible fluorescent lamps (cool white L36/W20, Osram) emitting 80 μmol m^−2^s^−1^ of PAR. A spectrum of the combination of lamps is found in Sommaruga and colleagues (1996).

Subsamples for DOC and absorbance were collected before and after exposure from all treatments. The samples were immediately filtered through a pre-combusted (4 h at 450°C) GF/F filter (Whatman), placed on a stainless steel syringe holder. Absorbance was measured in a spectrophotometer (double-beam Hitachi U-2000) from 250 nm to 750 nm using a 10 cm quartz cuvette (Suprasil I). Absorption coefficients at specific wavelengths (*a*_λ_) were calculated as *a*_λ_ = (2.303*D_λ_)/*L*, where *D*_λ_ is the absorbance at the wavelength considered and *L* the path length (m) of the cuvette. The coefficients were corrected for the effect of scattering by colloids using a long reference wavelength (740 nm).

DOC was measured by high temperature catalytic oxidation with a Shimadzu TOC analyser Model 5000. The instrument was equipped with a Shimadzu platinized-quartz catalyst for high sensitivity analysis. Three to five injections were analysed for every sample and blanks (Milli-Q water).

After exposure, the lake bacterial assemblage was inoculated in all treatments following a 1:10 dilution by filtering lake water collected at 2 m depth through a 0.8 μm polycarbonate membrane (ATTP, Millipore). The resulting bacterial cultures were incubated in the dark at 14°C for either 24 h (first experiment) or 48 h (second experiment). Subsamples for bacterial activity and abundance were collected at time 0 (0.5 h after the inoculation of the bacterial assemblage) and then every 24 h. During the second experiment, additional samples were removed to perform microautoradiography combined with fluorescent *in situ* hybridization and signal amplification by catalysed reporter deposition (MICRO-CARD-FISH).

### Incubation for microautoradiography

Subsamples were incubated with [^3^H]-l-leucine (Amersham, specific activity = 2331 GBq mmol^−1^; 20 nmol l^−1^ final concentration) at *in situ* temperature for 1 h. Incubation was ended by adding formaldehyde at a final concentration of 2%. Samples were fixed overnight at 4°C and filtered on the next day onto 0.22 μm polycarbonate white filters that were subsequently rinsed twice with 5 ml of particle-free Milli-Q water. Afterwards, filters were stored frozen (−20°C) until further processing.

### Bulk leucine incorporation

Leucine incorporation rates were estimated according to Simon and Azam (1989) in duplicate samples and one formaldehyde-killed control incubated with 20 nmol l^−1^ (final concentration) of [^3^H]-l-leucine (specific activity as previously stated). Samples (15–30 ml) were incubated at *in situ* temperature in the dark for 1 h. Incubations were terminated by adding formaldehyde at 2% final concentration. Subsequently, the samples were filtered through 0.22 μm Millipore GTTP filters and rinsed twice with 5 ml of 5% TCA for 5 min. The radioactivity of the filters was assessed after 15 h.

### Bacterial abundance

Bacterial numbers were assessed by flow cytometry. Subsamples of 450 μl were stained adding 25 μl of a 50 μmol l^−1^ SYTO 13 solution (Molecular Probes). Counts were made with a MoFlo (DakoCytomation) equipped with a water cooled argon-ion laser tuned at 488 nm (200 mW). Bacteria were detected by their signatures in a plot of orthogonal side scatter (SSC) versus green fluorescence (FL1).

### Hybridization and tyramide signal amplification

Fluorescent *in situ* hybridization with horseradish peroxidase (HRP)-labelled probes was carried out on filter sections according to Pernthaler and colleagues (2002), using a modified permeabilization protocol for freshwater bacteria (Sekar *et al*., 2003). Five different group-specific oligonucleotide probes (ThermoHybrid, Germany) were targeted to the domain *Bacteria* (EUB338; Amann *et al*., 1990), to *Betaproteobacteria* (BET42a; Manz *et al*., 1992) and its subgroup R-BT (R-BT065; Šimek *et al*., 2001), to *Cytophaga*-like bacteria (CF319a; Manz *et al*., 1996) and to the class *Actinobacteria* (HGC69a; Roller *et al*., 1994). The formamide concentration in the hybridization buffer was always 55% excepting for probe HGC69a which required 35% formamide.

### Microautoradiography

The procedure we used is described in detail elsewhere (Teira *et al*., 2004). Briefly, hybridized filter sections were transferred onto slides coated with a molten Kodak NTB emulsion. Subsequently, slides were placed on a cold plate for a few minutes until the emulsion hardened. Slides exposure was carried out at 4°C for 24 h in light-tight boxes containing a drying agent. Optimum exposure time was determined empirically in a preliminary experiment. Development and fixation of the slides were performed according to the specifications of the manufacturer. Afterwards cells were stained with an antifading solution containing DAPI to a final concentration of 1 μg ml^−1^ and the slides examined using a Zeiss Axioplan microscope equipped with a 100 W Hg lamp. Silver grains around bacterial cells were observed using the transmission mode of the instrument. Cells were counted in at least 20 randomly selected microscopic fields and for every field four different counts were recorded: (i) DAPI-positive cells, (ii) probe-specific positive cells, (iii) DAPI+ autoradiography positive cells and (iv) probe-specific + autoradiography-positive cells. At least 350 DAPI-stained cells were counted per sample.

### Statistical analysis

A two-way analysis of variance (factor A: exposure conditions, and factor B: type of DOM) was used to detect changes in the proportions of different bacterial groups (expressed as a percentage of DAPI-stained cells) present in the different cultures. This statistical analysis was also applied to detect differences in leucine incorporation rates and in the proportion of active cells in the different samples. The pertinent *post hoc* comparisons were made by the Holm–Sidak method with an overall significance level of 0.05.
